# Neuroskeletal Effects of Chronic Bioelectric Nerve Stimulation in Health and Diabetes

**DOI:** 10.3389/fnins.2021.632768

**Published:** 2021-04-07

**Authors:** Alec T. Beeve, Ivana Shen, Xiao Zhang, Kristann Magee, Ying Yan, Matthew R. MacEwan, Erica L. Scheller

**Affiliations:** ^1^Department of Biomedical Engineering, Washington University in St. Louis, St. Louis, MO, United States; ^2^Department of Internal Medicine, Division of Bone and Mineral Diseases, Washington University School of Medicine in St. Louis, St. Louis, MO, United States; ^3^Department of Neurosurgery, Washington University School of Medicine in St. Louis, St. Louis, MO, United States

**Keywords:** bone, nerves, neuropathy, electrical stimulation, muscle, type 1 diabetes (T1D), gait, bone marrow adiposity

## Abstract

**Background/Aims:**

Bioelectric nerve stimulation (eStim) is an emerging clinical paradigm that can promote nerve regeneration after trauma, including within the context of diabetes. However, its ability to prevent the onset of diabetic peripheral neuropathy (DPN) has not yet been evaluated. Beyond the nerve itself, DPN has emerged as a potential contributor to sarcopenia and bone disease; thus, we hypothesized that eStim could serve as a strategy to simultaneously promote neural and musculoskeletal health in diabetes.

**Methods:**

To address this question, an eStim paradigm pre-optimized to promote nerve regeneration was applied to the sciatic nerve, which directly innervates the tibia and lower limb, for 8 weeks in control and streptozotocin-induced type 1 diabetic (T1D) rats. Metabolic, gait, nerve and bone assessments were used to evaluate the progression of diabetes and the effect of sciatic nerve eStim on neuropathy and musculoskeletal disease, while also considering the effects of cuff placement and chronic eStim in otherwise healthy animals.

**Results:**

Rats with T1D exhibited increased mechanical allodynia in the hindpaw, reduced muscle mass, decreased cortical and cancellous bone volume fraction (BVF), reduced cortical bone tissue mineral density (TMD), and decreased bone marrow adiposity. Type 1 diabetes also had an independent effect on gait. Placement of the cuff electrode alone resulted in altered gait patterns and unilateral reductions in tibia length, cortical BVF, and bone marrow adiposity. Alterations in gait patterns were restored by eStim and tibial lengthening was favored unilaterally; however, eStim did not prevent T1D-induced changes in muscle, bone, marrow adiposity or mechanical sensitivity. Beyond this, chronic eStim resulted in an independent, bilateral reduction in cortical TMD.

**Conclusion:**

Overall, these results provide new insight into the pathogenesis of diabetic neuroskeletal disease and its regulation by eStim. Though eStim did not prevent neural or musculoskeletal complications in T1D, our results demonstrate that clinical applications of peripheral neuromodulation ought to consider the impact of device placement and eStim on long-term skeletal health in both healthy individuals and those with metabolic disease. This includes monitoring for compounded bone loss to prevent unintended consequences including decreased bone mineral density and increased fracture risk.

## Introduction

Therapeutic use of electrical stimuli, or bioelectric medicine, is ancient. For centuries, humans have exploited the responses of the body to electrical stimuli for medical treatment, starting with pain and expanding to increasingly complex disorders from hearing loss to paralysis (Chang, 2021). Bioelectric medicine has also provided solutions to musculoskeletal disease and injury. For example, electrical current delivered to nearby bone fractures can enhance healing outcomes (Aleem et al., 2016), and long, thin electrodes implanted near the spine or peripheral nerves can relieve chronic back or joint pain (Kapural et al., 2016; Ilfeld et al., 2019; Deer et al., 2021). Additionally, electrical stimulation of peripheral nerves (eStim) offers a unique opportunity to utilize the interconnectedness and regulatory function of the nervous system to treat diverse conditions throughout the body. For example, one of the most widely applied therapeutics in bioelectric medicine today is vagus nerve stimulation. Promising results have been shown for both rheumatoid arthritis and obesity in using eStim to harness the anti-inflammatory and satiety-mediating functions of the vagus nerve (Koopman et al., 2016; Apovian et al., 2017).

In the past two decades, eStim paradigms that enhance the regenerative capacity of nerves in rodents and humans post-injury have also been established. In rats and mice, both motor and sensory nerves demonstrate upregulated regeneration-associated genes with application of eStim (Brushart et al., 2005; Geremia et al., 2007; Gordon and English, 2016). In humans, one application of post-surgical eStim similarly improves functional nerve regeneration after repair of digital nerve transection and median nerve crush injury (i.e., carpal tunnel syndrome) (Gordon et al., 2010; Wong et al., 2015). Studies in mice and rats suggest that the neuroregenerative effects of eStim post-injury persist even in the metabolically challenged state of streptozotocin (STZ)-induced type 1 diabetes (T1D) (Lin et al., 2015; Singh et al., 2015).

In this study, we hypothesized that a neuroregenerative eStim paradigm may be sufficient to halt the progression of diabetic peripheral neuropathy (DPN), contributing to restoration of function. Considering correlations between neural and musculoskeletal health in diabetes (Melendez-Ramirez et al., 2010; Forbes and Cooper, 2013; Jaiswal et al., 2017; Beeve et al., 2019), we also hypothesized that eStim would provide a simultaneous benefit for innervated downstream organs, including muscle and bone, in both control and diabetic rats. To test this hypothesis, we utilized a fully implantable, wireless system for sciatic nerve stimulation that has previously been employed to promote nerve regeneration (MacEwan et al., 2018). This technology enabled us to deliver weekly 1 h eStim treatments for 8 weeks with a silicone nerve cuff electrode. Coupled with metabolic, neural and musculoskeletal analyses, our experimental design allowed us to determine the *in vivo* effect of chronic eStim on nerve, muscle and bone in the context of health and diabetes. This work was completed as part of the National Institutes of Health SPARC consortium (Stimulating Peripheral Activity to Relieve Conditions) in the United States.

## Materials and Methods

### eStim Device Fabrication

**FIGURE 1:**
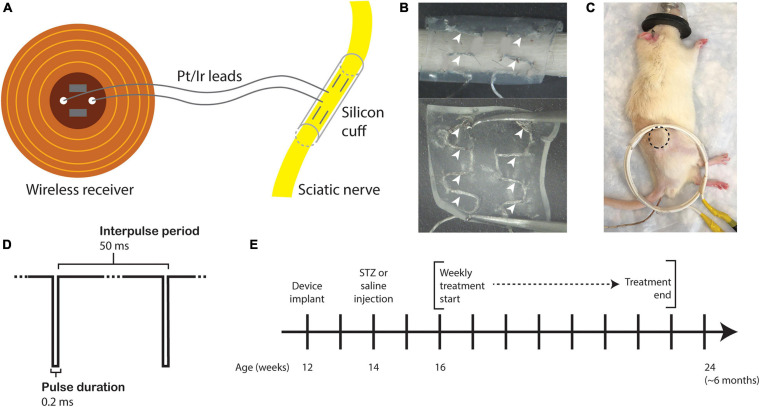
Study design. **(A)** Drawing of the sciatic nerve cuff stimulator. **(B)** Outer surface (top) and inner surface (bottom) of the silicone cuff threaded with Pt/Ir electrode leads. White arrowheads indicate horizontally threaded regions in which the electrode wire is exposed to the nerve. **(C)** Rat under anesthesia for eStim treatment with location of implanted receiver indicated (dotted circle) with external power-transmitting coil (white coil). A rectal probe was inserted for thermal monitoring (wire under tail). **(D)** Stimulus waveform. **(E)** Experimental timeline.

Fully implantable sciatic nerve cuff stimulators were fabricated as previously described (MacEwan et al., 2018). Braided Pt/Ir leads (10IR9/49T, Medwire, Sigmund Cohn Corp.) were threaded through 8 mm of silicone tubing (inner diameter 1.5 mm) using a custom plastic rig (Figures 1A,B). Exposed wires on the external surface of the silicone cuff were insulated with medical-grade silicone elastomer (A-564, Factor II). Cuff leads were then soldered to a thin-film wireless receiver coil and embedded in the same medical silicone (Figure 1A). As the average diameter of the rat sciatic nerve is approximately 1 mm (Isaacs et al., 2014; Onode et al., 2019), it was neither expected nor intended that the cuff would constrict the sciatic nerve at any stage of development.

### Animals

The procedures in this investigation were approved by the Washington University Animal Studies Committee (Saint Louis, MO, United States). Male Lewis rats (7 weeks, Strain 004) were obtained from Charles River and housed on a 12-hour light/dark cycle while fed *ad libitum* (LabDiet, 53WU, PicoLab Rodent Diet 20).

### eStim Device Implantation

All surgeries were conducted under anesthesia with 2% isoflurane. All animals were implanted unilaterally with a device onto the right side at 12-weeks of age. An incision was made on the lateral surface of the right thigh to expose the sciatic nerve. The silicone cuff was placed around the nerve and closed with one stitch of 6-O nylon suture (McKesson, REF S1698GX), and the receiver was placed subcutaneously proximal to the cuff (Figure 1C, dotted circle). Device function was verified at the time of surgery by activating the device with a superimposed transmitter coil (Figure 1C, white coil) and the implant site muscle and skin layers were closed with 5-O Vicryl (Ethicon, J303H) and 4-O Nylon (McKesson, REF S662GX) suture, respectively. Animals received a single 1.0 mg/kg dose of buprenorphine sustained-release (ZooPharm) subcutaneously 1 h before surgery for post-operative analgesia.

### Induction of Diabetes

Type 1 diabetes was induced in rats at 14-weeks of age, 2 weeks before beginning eStim treatments. A 2 week delayed-onset treatment paradigm was selected to allow for early progression of disease, mirroring clinical intervention for diabetic complications such as neuropathy and bone loss that begins after diagnosis of disease and/or onset of symptoms. Animals were fasted for 24 h on aspen bedding, after which a single 65 mg/kg dose of STZ was administered by IP injection. Controls received saline vehicle. After injection, fasting was continued for 2 h prior to returning food and 10% sucrose filter-sterilized water was provided *ad libitum* for 24 h. After 24 h, the rats were returned to cobb bedding and filtered water. Body mass and tail blood glucose were monitored daily after STZ injection with an electronic scale and with a tail prick by blood glucometer (Bayer Contour Next) for 3 days post-injection. Blood glucose and body mass were subsequently recorded weekly for all animals. Rats exhibiting successive reductions in weight and bradykinesia were given biweekly 2 mL subcutaneous saline injections, wet food and hydrogel until stable body mass was restored.

### eStim Treatment Regimen

Beginning at 16-weeks of age and continuing for 8 weeks total, those animals assigned to the stimulation group were treated weekly for 1 h with a supramaximal cathodic square pulse, 0.2 ms pulse duration, and 20 Hz frequency (Figure 1D). Animals were anesthetized with 2% isoflurane and placed on a heating pad throughout treatment. These parameters were previously shown to enhance neural regrowth post-transection (Geremia et al., 2007; MacEwan et al., 2018). Sham animals received only anesthesia, also weekly for 1 h. Body temperature was monitored in all animals with a rectal thermometer probe (Extech Instruments Easy View 10, Figure 1B, wire under tail). Treatments began 2 weeks post-STZ and continued for 8 weeks prior to end point analysis at 24 weeks of age (Figure 1E). There were four experimental groups in this study: control sham, control eStim, T1D sham, and T1D eStim.

At the start of the experiment, all animals showed maximal activation of muscles in the lower limb at a stimulation amplitude of 8–9 V. Maximal muscular activation was defined visually by pointed toes and clenched paw and by palpating twitches in the tibialis anterior and gastrocnemius muscles (Supplementary Video 1). Upon implantation of a neuroprosthetic device, it was expected that some fibrotic encapsulation would occur that might affect the magnitude of muscle activation over time. Therefore, muscular contraction was assessed visually and by palpation during each week of eStim for each animal to ensure that the stimulations achieved this maximally activated effect. If the effect was not achieved by the initial stimulus intensity, it was increased by raising first the voltage and then the pulse duration in order to maintain the maximally activated muscle contraction observed at the start of the experiment.

### Single Frame Motion Analysis

Single frame motion analysis (SFMA) was performed at 10- and 23-weeks of age. Animals were trained to walk across a 3-foot-long, 3.5-inch wide wooden plank to their home cage by placing them on the plank at increasingly distant positions from their home cage. Tickling was used as a reward for task completion (Cloutier et al., 2018). Once animals were adequately trained to walk from the most distant end of the plank to their home cage without pausing, the animals were recorded from behind (iPhone X). Video analysis was performed in MATLAB using a custom guided-user interface designed to measure the first-quadrant angle between the horizontal axis and the foot-to-base vector – this parameter is called the foot-to-base angle (FBA) (Fey et al., 2010). The horizontal axis was determined by the edge of the home cage. The foot-to-base vector was measured by drawing a line between the heel and the midplantar surface at a frame just before pushoff. The angle was measured for every frame available for each foot while the animal was actively walking.

### Von Frey

The up-down method was used to assess mechanical allodynia in all animals at 23-weeks of age (Chaplan et al., 1994). Animals were placed on a chicken-wire metal grid fixed to a wooden frame. The frame was elevated 1.5-feet above a countertop to allow for testing and viewing the plantar surface of the paw. A mouse cage was placed over the animal to restrain motion. Animals were allowed to acclimate for 5 min, at which point they were no longer actively exploring their environment. Manual von Frey monofilaments ranged from 8 to 300 g force. A response was recorded as hindpaw withdrawal upon or subsequent to application of the filament. Filaments were applied on alternating sides as described previously, and after a response, animals were allowed to reacclimate for 1 min before the applying the next filament.

### *In vivo* Computed Tomography: Cortical Bone

All animals were scanned *in vivo* at 12- and 24-weeks of age. Animals were anesthetized with 1–2% isoflurane and placed into the scanning bed. A piece of VetWrap bandage was taped over the animal’s torso to reduce loss of body heat. The top limb was placed into a rig to straighten and stabilize the leg during the scan with the foot secured. *In vivo* scans were conducted on the mid-diaphysis: a 3 mm region (200 slices) centered halfway between the proximal end of the tibia and the tibiofibular junction (VivaCT40; Scanco Medical; 70 kVp; 114 uA; 15 μm voxel size). After the first limb was scanned, the animal was rotated on the bed and the scan process was repeated for the contralateral limb. The total time under anesthesia exceeded no more than 1 h. Analysis was performed using the Scanco software. The entire 3 mm ROI at the mid-diaphysis was contoured and analyzed at a threshold of 250 with sigma and support values of 0.8 and 1, respectively. Bone volume fraction (BV/TV), cortical thickness (mm), tissue mineral density (mg HA/cm^3^), total area (mm^2^), bone area (mm^2^), medullary area (mm^2^), and pMOI (mm^4^) were extracted for data analysis.

### *Ex vivo* Computed Tomography: Cancellous Bone

Prior to sectioning for histology, explanted tibias were embedded in 2% agarose, and a 6 mm region was scanned starting at the growth plate (VivaCT40; Scanco Medical; 70 kVp; 114 uA; 15 μm voxel size). Analysis was performed using the Scanco software. From 2 mm (133 slices) below the growth plate, an ROI of 1.5 mm (100 slices) was contoured and analyzed at a threshold of 240 with 0.8 sigma and 1 support. Total volume (mm^3^), bone volume (mm^3^), bone volume fraction (BV/TV), structural model index (SMI), connectivity density, trabecular number, trabecular thickness (mm), trabecular separation (mm), and tissue mineral density (mg HA/cm^3^) were extracted for analysis.

### Histology and Bone Marrow Adipocyte Analysis

At the end point (24 weeks), rats were euthanized *via* carbon dioxide overdose followed by pneumothorax. Tissues were collected and weighed on an electronic scale. Collected tissues were fixed in 10% neutral buffered formalin (Fisher Scientific 23-245684) for 24 h prior to processing as detailed below. Tibia length was measured with digital calipers (iKKEGOL).

All histology was performed by the WUSM Musculoskeletal Histology and Morphometry core. Prior to embedding, tibias were dehydrated in a reverse gradient to 70% ethanol. Tibias were bisected transversely at the 50% site between the proximal end and the tibiofibular junction and approximately 2 mm below the growth plate to achieve cross-sections corresponding to our regions of CT analysis. Bones were fully decalcified in 14% EDTA (Sigma-Aldrich E5134), pH 7.4 prior to paraffin embedding, sectioning (10 μm thickness) and staining with hematoxylin and eosin. Images were taken on a Hamamatsu 2.0-HT Nano Zoomer System with NDP.scan 2.5 image software at 20× in bright field mode.

The acquired images were exported as TIFF files under 10× magnification and were processed in Fiji to measure average adipocyte cell size and number. Briefly, the scale in Fiji was first set to be consistent with the original image (1.084 pixels/μm). The image was then converted to 8-bit and the cortical bone was specifically selected by thresholding. A median filter with a radius of 10 pixels was applied. The image was then inverted and the area of bone marrow cavity was selected and measured using the “Wand tool” and the “Measure” command. A threshold of 230 to 255 was applied to the original 8-bit image and everything outside the bone marrow cavity was cleared using the “Clear Outside” command. A median filter with a radius of two pixels was applied to the image and the non-adipocyte structures were selected and eliminated using the “Analyze Particles” tool by setting the circularity to 0–0.2. The cleaned image was further processed using the “Watershed” tool and the adipocyte size and number were finally determined using the “Analyze Particles” tool by setting the size to 200 to 4000 μm^2^ and circularity to 0.50–1.00. The average adipocyte cell size and number per bone marrow area were calculated using Excel.

### Statistics

Statistical analyses for this study were performed in GraphPad Prism. Tests included two-way ANOVA, three-way ANOVA, and mixed effects analyses. Specific information on statistical tests is detailed in the figure legends. A *p*-value of less than 0.050 was considered statistically significant. Quantitative assessments including bone length, organ mass, microcomputed tomography, behavioral assessments, and bone marrow adiposity measurements were performed by individuals blinded to the experimental groups.

## Results

### Regulation of Blood Glucose, Body, and Tissue Mass by T1D and eStim

**FIGURE 2:**
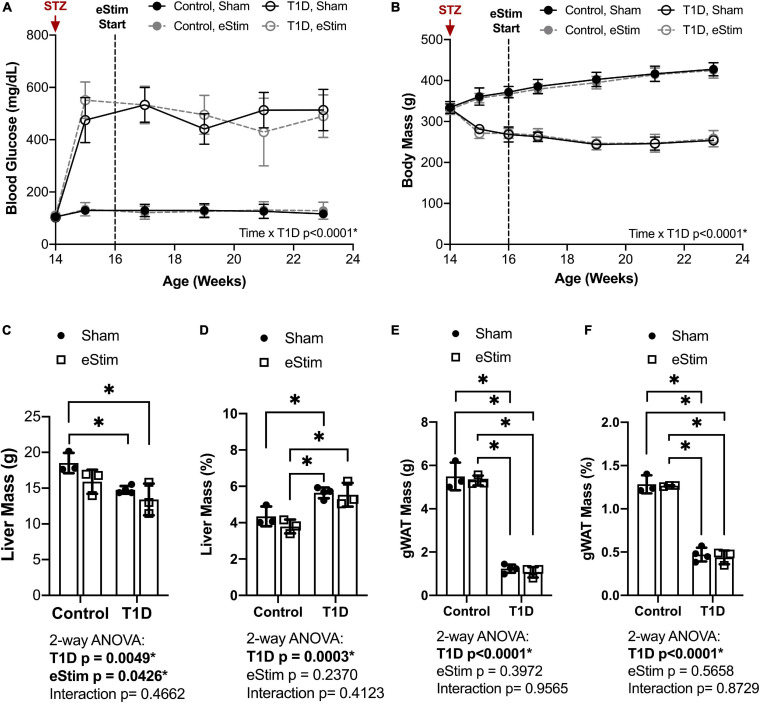
Blood glucose and body and organ mass. Blood glucose and body mass were evaluated starting at the time of STZ induction (14-week of age) up to the final week of treatment (24-week of age). **(A)** Blood glucose. **(B)** Body mass. **(C)** Liver mass at end point. **(D)** Liver mass normalized to body mass. **(E)** Gonadal white adipose tissue (gWAT) mass at end point. **(F)** gWAT mass normalized to body mass. Statistics for blood glucose and body mass were performed by mixed effects and three-way ANOVA analyses, respectively, as a single missing value was present in the blood glucose data; Control, sham *n* = 7; Control, eStim *n* = 6; Diabetic, sham *n* = 8; Diabetic, eStim *n* = 5; *p < 0.050. Statistics for organ masses was performed by two-way ANOVA with Sidak’s multiple comparisons test; Control, sham *n* = 3; Control, eStim *n* = 3; Diabetic, sham *n* = 4; Diabetic, eStim *n* = 3; *p < 0.050.

Blood glucose and body mass were monitored longitudinally to confirm diabetic induction and sustained hyperglycemia throughout the study. In T1D rats, blood glucose increased from 106 ± 7 to 503 ± 70 mg/dL post-injection with STZ (Figure 2A). Sustained hyperglycemia was maintained throughout the course of the experiment (Figure 2A). By contrast, control animals remained normoglycemic (Figure 2A). From the time of T1D onset at 14-weeks of age to the final week of treatment, body mass increased by 28 ± 4% in control animals and decreased by 23 ± 4% in diabetic animals relative to baseline (Figure 2B). As expected, unilateral sciatic nerve eStim for 1 h per week did not influence body mass or blood glucose in healthy or diabetic animals, relative to sham, anesthesia-only controls (Figures 2A,B).

At the end point, tissues including liver, gonadal white adipose tissue (gWAT), and spleen were dissected and weighed to gauge overall health. Type 1 diabetes resulted in reduced absolute liver and gWAT mass (Figures 2C,E). When normalized to body weight, liver mass was elevated by 37% in T1D rats (Figure 2D) and the relative quantity of gWAT was reduced by 78% (Figure 2F). Bioelectric nerve stimulation resulted in reduced absolute liver mass, but normalization to body weight eliminated this effect (Figures 2C,D). Bioelectric nerve stimulation did not alter gWAT (Figures 2E,F). Type 1 diabetes caused absolute decreases in spleen mass that were proportional to body size and not impacted by eStim (data not shown). Overall, this confirmed that intermittent, unilateral stimulation of the sciatic nerve did not cause overt global changes in peripheral tissues.

**FIGURE 3:**
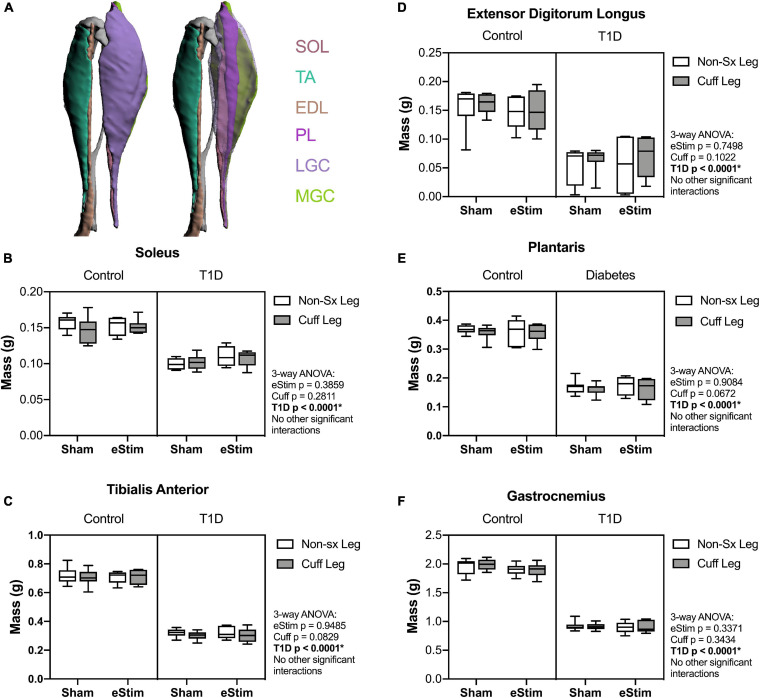
Muscle mass. At end point, muscles surrounding the tibia **(A)** were dissected and weighed. **(B)** Soleus (SOL) muscle mass. **(C)** Tibialis anterior (TA) muscle mass. **(D)** Lateral and medial gastrocnemius (LGC, MGC) combined muscle mass. **(E)** Extensor digitorumlongus (EDL) muscle mass. **(F)** Plantaris (PL) muscle mass. Control, sham *n* = 7; Control, eStim *n* = 6; Diabetic, sham *n* = 8; Diabetic, eStim *n* = 5; three-way ANOVA with repeated measures (Non-sx Leg vs. Cuff Leg); *p < 0.050. Panel A generated from Charles et al. (2016).

Stimulation of the sciatic nerve causes unilateral muscle contraction (Supplementary Video 1). We hypothesized that this may be sufficient to increase muscle mass in healthy animals and to rescue muscle atrophy in those with T1D. In previous studies, type II (fast-twitch) muscle fibers and type I (slow-twitch) fibers were differentially regulated by T1D in the STZ-induced model (Rutschmann et al., 1984; Cotter et al., 1989). Thus, muscles of predominately type I [soleus (SOL)], type II [tibialis anterior (TA), extensor digitorum longus (EDL), plantaris (PL)] and mixed type fiber compositions [lateral and medial gastrocnemius (LGC/MGC)] were selected for analysis (Figure 3A). Rats with T1D had reduced hindlimb muscle mass relative to controls (Figures 3B–F). Specifically, in diabetic animals, muscle masses were bilaterally reduced by 32% in the soleus (Figure 3B); by 56, 60, and 54% in the tibialis anterior, EDL, and plantaris, respectively (Figures 3C–E); and by 53% in the gastrocnemius (Figure 3F). Consistent with previous reports (Rutschmann et al., 1984; Cotter et al., 1989), muscles containing a significant population of type II fibers were more severely affected by diabetes than muscles with predominately type I fibers. In addition to the effect of diabetes, we also considered the effects of sciatic nerve cuff placement and eStim using three-way ANOVA (T1D × Cuff × eStim). The placement of a sciatic nerve cuff did not independently influence muscle mass. In addition, contrary to our expectations, eStim treatment did not significantly alter or improve muscle mass in control rats or those with T1D (Figures 3B–F).

### Gait Alterations and Mechanical Allodynia With T1D, Sciatic Nerve Cuff, and eStim

**FIGURE 4:**
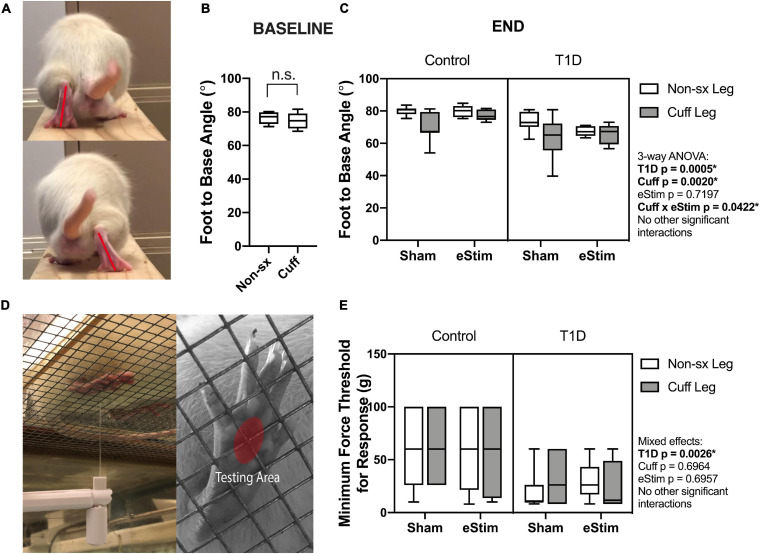
Neuromuscular behavioral assessments. **(A)** Foot to base angle (FBA) was measured using single frame motion analysis (SFMA). **(B)** Baseline FBA measurements at 10 weeks of age. **(C)** End point FBA measurements at 23-week of age. Manual von Frey filaments were also applied to the midplantar surface of the hindpaw (indicated by a red circle) **(D)**. **(E)** Minimum force threshold for response at end point. Statistics for baseline FBA measurements was performed by paired *t*-test. Control, sham *n* = 7; Control, eStim *n* = 6; Diabetic, sham *n* = 8; Diabetic, eStim *n* = 5; three-way ANOVA with repeated measures (Non-sx Leg vs. Cuff Leg); *p < 0.050.

As the sciatic nerve is involved in locomotion, its manipulation could result in gait changes. For example, FBA is reduced in rodents with sciatic nerve damage (Fey et al., 2010). To assess this, single-frame motion analysis was conducted at 10- and 23-weeks of age. Foot-to-base angle was measured as the first-quadrant angle between the line from midplantar surface to heel and the perpendicular (home cage edge) (Figure 4A; Fey et al., 2010; Kruspe et al., 2014). At baseline, prior to surgery, FBA was not significantly different between the left and right limbs (Figure 4B). At the end point, T1D resulted in a bilateral reduction of FBA by −12%, independent of cuff placement or eStim (Figure 4C). Foot-to-base angle was further reduced unilaterally on the cuffed side of non-stimulated animals by −12 ± 10% in controls and by −13 ± 14% in diabetics (Figure 4C). In stimulated animals, however, this effect was suppressed. The FBA of the cuffed limb was partially restored in control and diabetic eStim groups to −3 ± 7 and −3 ± 13%, respectively, relative to the control side (Figure 4C). Overall, these results show a unilateral, detrimental effect of sciatic nerve cuffing on gait that is partially restored by eStim.

Rodent models of T1D exhibit mechanical allodynia, representative of sensory DPN (Morrow, 2004). We hypothesized that application of neuroregenerative eStim would oppose the progression of DPN, resulting in normalization of mechanical sensitivity. Cuff placement can also independently cause unilateral sensitivity if placed too tightly around the nerve (Mosconi and Kruger, 1996; Austin et al., 2012). Thus, we measured mechanical allodynia at 23-weeks of age (one week before end point) using manual von Frey filaments applied to the mid-plantar surface of the hindpaw [Figure 4D, red oval; (Chaplan et al., 1994)]. As expected, diabetic animals exhibited, on average, a 56% reduction in the response threshold bilaterally compared to controls, indicative of increased sensitivity to mechanical stimuli (Figure 4E, three-way ANOVA, T1D *p* = 0.0026). However, contrary to our expectations, eStim treatment did not alter the mechanical sensitivity of the controls or rescue the mechanical allodynia of those with T1D (Figure 4E, three-way ANOVA, eStim *p* = 0.6957). The presence of the unilateral sciatic nerve cuff also did not impact the mechanical sensitivity in the cuffed limb in either control or diabetic animals, confirming the absence of overt nerve constriction or irritation in our model (three-way ANOVA, Cuff *p* = 0.6964).

### Changes in Bone and Bone Marrow Adiposity With T1D, Sciatic Nerve Cuff, and eStim

#### Bone Length

**FIGURE 5:**
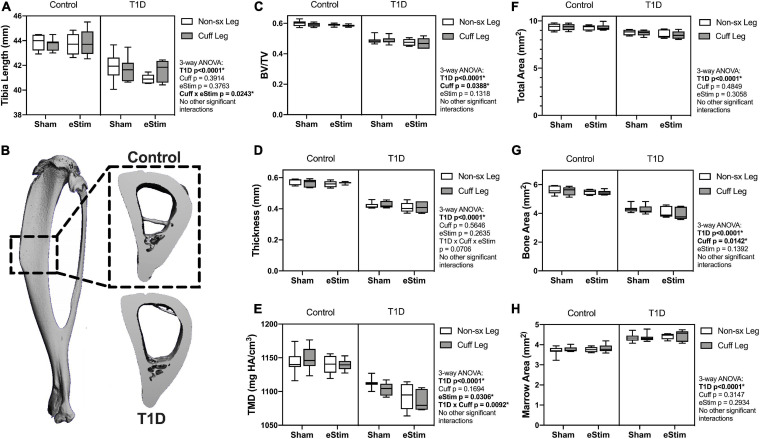
Tibia length and cortical bone. **(A)** End point tibial length as assessed after dissection using digital calipers. Cortical bone was evaluated at 24-week of age by μCT in a 3 mm region centered midway between the proximal end of the tibia and the tibia-fibula junction **(B)**. **(C)** Cortical bone volume fraction (BV/TV). **(D)** Cortical thickness. **(E)** Cortical tissue mineral density (TMD). **(F)** Cortical total area. **(G)** Cortical bone area. **(H)** Cortical marrow area. Control, sham *n* = 7; Control, eStim *n* = 6; Diabetic, sham *n* = 8; Diabetic, eStim *n* = 5; three-way ANOVA with repeated measures (Non-sx Leg vs. Cuff Leg); *p < 0.050.

Sensory neurotransmitters such as calcitonin gene related peptide (CGRP) have previously been shown to promote bone formation in developing animals (Xu et al., 2020). By contrast, T1D can limit bone growth (Silva et al., 2009). We hypothesized that chronic nerve activation would promote bone formation in both control and T1D animals due to increased local release of anabolic neurotransmitters and activation of muscle contraction. To assess the effects of T1D, cuff placement, and eStim on skeletal growth, left and right side tibial lengths were measured with digital calipers and compared using three-way ANOVA (T1D × Cuff × eStim). Tibial length was decreased bilaterally by 5% in rats with T1D when compared to controls, independent of cuff placement or eStim (Figure 5A, T1D *p* < 0.0001). In addition, the cuffed limb was slightly shorter than the non-surgical limb in sham-treated animals (−0.3 and −0.5% in control and diabetic animals, respectively; Figure 5A). By contrast, in animals treated with unilateral chronic eStim the cuffed limb was slightly longer (+0.3 and +1.6% in control and diabetic animals, respectively) (Figure 5A), indicating an interaction between sciatic nerve cuffing and eStim on longitudinal bone growth (three-way ANOVA, Cuff × eStim *p* = 0.0243).

#### Cortical Bone

As mentioned above, sensory nerves in bone are thought to release anabolic neuropeptides near bone-forming cells (Tomlinson et al., 2016; Brazill et al., 2019); thus, we expected that DPN prevention and/or neural activation by eStim would increase bone mass. Cortical bone was analyzed by *in vivo* microcomputed tomography at 12-weeks (baseline) and 24-weeks of age (end point) in a 3 mm section of the mid-diaphysis, centered between the proximal end of the tibia and the tibia-fibula junction (Figure 5B). At baseline, prior to cuff implantation, the left and right limbs exhibited no difference in bone size or morphology (data not shown), although cortical TMD was 0.8% lower, on average, in the right limb (the limb to be cuffed) relative to the left at baseline (*p* = 0.0412).

At end point, rats with T1D demonstrated a 19% reduction in cortical bone volume fraction (BVF), a 26% decrease in cortical thickness, and a 4% decrease in cortical TMD relative to non-diabetic controls (Figures 5C–E). Reduced bone quantity in diabetic animals was driven by a 24% reduction in bone area, a 7% decrease in total area, and a 16% increase in marrow area (Figures 5F–H). Sciatic nerve cuffing over 12 weeks had a small negative effect on cortical BVF in the cuffed limb (ranging from −0.4 to −1.5%), independent of eStim or T1D (Figure 5C). This effect was also reflected by a unilateral 1 to 3% reduction in bone area in the cuffed limb relative to the non-surgical control side (Figure 5G). The only observed effect of eStim on cortical bone was a 0.6 and 1.6% bilateral reduction in TMD in control and diabetic animals, respectively, relative to non-stimulated, sham controls (Figure 5E). In summary, T1D resulted in reduced cortical bone quantity and mineral density that was not rescued by eStim. In fact, eStim and sciatic nerve cuffing introduced additional cortical bone deficits in both control and diabetic animals including bilaterally reduced TMD (as a result of eStim) and unilaterally reduced bone mass (as a result of sciatic nerve cuffing).

#### Cancellous Bone

**FIGURE 6:**
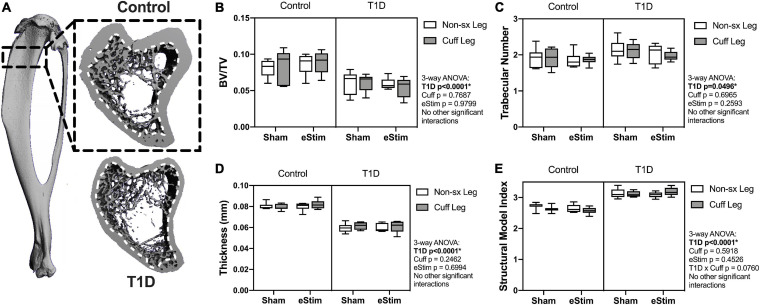
Cancellous bone. Cancellous bone was analyzed 2 mm below the growth plate in a 1.5 mm region **(A)**, dotted line represents contour inside of which bone was analyzed. **(B)** Cancellous bone volume fraction (BV/TV). **(C)** Trabecular number. **(D)** Trabecular thickness. **(E)** Structural model index (SMI). Control, sham *n* = 7; Control, eStim *n* = 6; Diabetic, sham *n* = 8; Diabetic, eStim *n* = 5; three-way ANOVA with repeated measures (Non-sx Leg vs. Cuff Leg); *p < 0.050.

Long bones are filled with spongy, cancellous bone that is concentrated largely at the metaphyses. This bone has a high turnover rate and is susceptible to systemic change, including well-documented decreases in rodents with T1D (Silva et al., 2009). To assess the effects of T1D, cuff placement and eStim on metaphyseal cancellous bone, we analyzed a 1.5 mm region starting 2 mm below the growth plate (Figure 6A). Consistent with previous reports, cancellous BVF was reduced by 31% in rats with T1D (Figure 6B). This was associated with a 10% increase in trabecular number and a 25% decrease in trabecular thickness (Figures 6C,D). Animals with T1D exhibited a trending 20% reduction in connectivity density (data not shown; three-way ANOVA, *p* = 0.0651) and a significant increase in structure model index (SMI) (Figure 6E; 2.6 vs. 3.1; SMI = 0 for plates, 3 for rods and 4 for solid spheres). Cancellous bone mineral density (BMD) was not significantly different between groups (data not shown). Unlike the effects observed with T1D, cancellous bone quantity, morphology, and mineralization were not modified by sciatic nerve cuffing or chronic eStim.

#### Bone Marrow Adiposity

**FIGURE 7:**
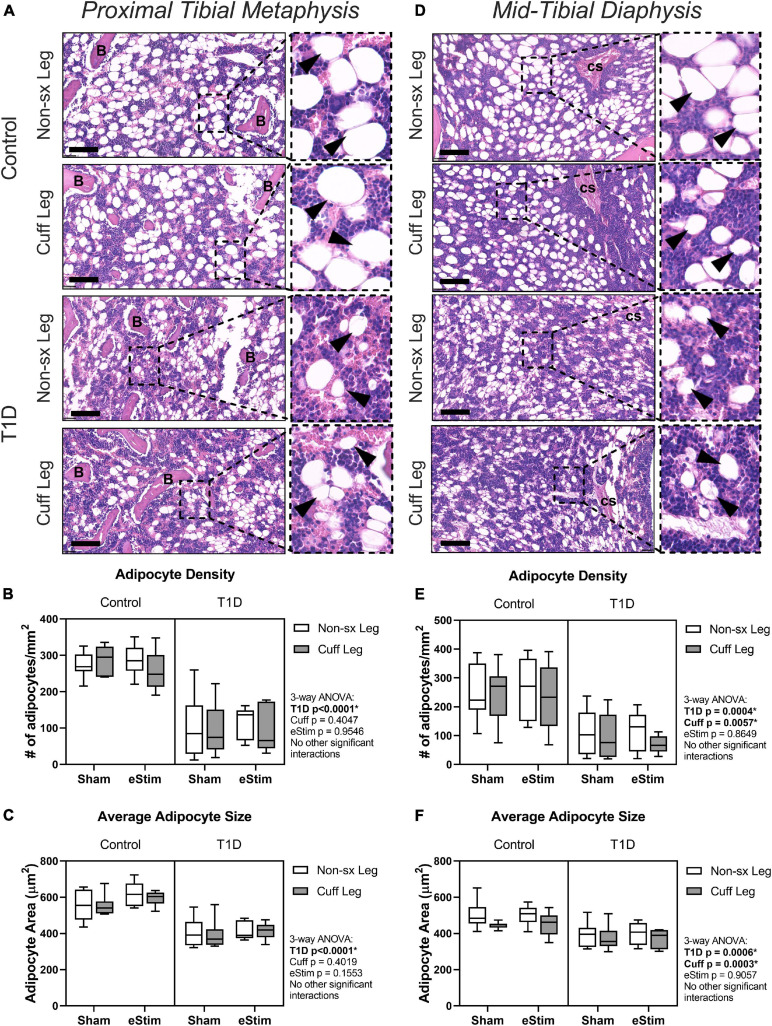
Bone marrow adipose tissue. Bone marrow adiposity was analyzed in the metaphysis 2 mm below the growth plate and in the mid-diaphysis. **(A)** BMAT histology in the metaphysis in healthy and control animals. **(B)** Adipocyte density in the metaphysis. **(C)** Average adipocyte size in the metaphysis. **(D)** BMAT histology in the diaphysis in health and control animals. **(E)** Adipocyte density in the diaphysis. **(F)** Average adipocyte size in the diaphysis. B = bone; cs = central venous sinus. Scale = 100 μm. (Control, sham *n* = 7; Control, eStim *n* = 6; Diabetic, sham *n* = 8; Diabetic, eStim *n* = 5) three-way ANOVA with repeated measures (Non-sx Leg vs. Cuff Leg); *p < 0.050.

In addition to bone, the skeleton is filled with a unique population of adipocytes that are collectively known as the bone marrow adipose tissue (BMAT). Bone marrow adipose tissue is an emerging regulator of hematopoietic, metabolic and skeletal health (Scheller et al., 2016). The ability of nerve stimulation to regulate bone marrow adipocytes *in vivo* remains unknown. In this study, bone marrow adipocytes were analyzed in the metaphysis approximately 2 mm below the growth plate (Figures 7A–C) and in the diaphysis approximately 2 mm above the peak of the tibial crest (Figures 7D–F), matching our regions of skeletal morphologic assessment in Figures 5, 6, respectively. Type 1 diabetes and sciatic nerve cuff placement both had independent effects on BMAT (Figure 7); however, the effect of sciatic nerve cuffing was region-specific. In the metaphysis, T1D resulted in a 59% reduction in BMA number, a 63% decrease in BMA density, and a 30% reduction in BMA size (Figures 7A–C and data not shown). Neither sciatic nerve cuffing nor eStim resulted in a change in metaphyseal BMAT. In the diaphysis, T1D similarly, resulted in a 39% reduction in BMA number, a 59% decrease in BMA density, and a 19% reduction in BMA size (Figures 7D–F and data not shown). However, contrary to the metaphyseal data, sciatic nerve cuffing alone also caused a 5–32% reduction in BMA number, a 5–38% reduction in BMA density, and a 4–12% reduction in adipocyte cell size relative to the contralateral limb (Figures 7D–F and data not shown). These cuff-side effects on bone marrow adiposity were driven primarily by changes in the non-diabetic control group and align spatially with previously reported differences in the innervation of the bone marrow adipocyte population along the length of the limb (Wee et al., 2019). Bioelectric stimulation did not modify the effects of T1D or cuff placement on bone marrow adiposity.

## Discussion

Despite promising results showing the pro-regenerative benefits of eStim (Geremia et al., 2007; Wong et al., 2015; Gamble et al., 2016), studies to date have not addressed its utility to prevent the onset of neuropathic symptoms and musculoskeletal decline in the context of metabolic diseases like T1D. Our experimental design allowed us to address the above questions using a fully implantable, wireless sciatic nerve stimulation device in a rat model of T1D while also considering the effects of cuff placement and chronic eStim in otherwise healthy animals.

Rodent models of T1D are characterized by progressive increases in mechanical sensitivity in addition to deficits in bone quantity, muscle mass and neuromuscular function (Cotter et al., 1989; Morrow, 2004; Silva et al., 2009). Similar to previous studies, we observed a significant decrease in hindlimb muscle mass, reduced withdrawal threshold to mechanical stimuli in the hindpaw, changes in gait, and substantial decreases in bone in animals with T1D. These results are entirely consistent with previous studies with one exception. Previous reports of STZ-induced T1D in rodents have demonstrated an increase in bone marrow adiposity and a decrease in peripheral fat mass (Botolin et al., 2005; Martin and McCabe, 2007). While we did observe a significant reduction in peripheral fat, we did not observe an increase in tibial bone marrow adipocyte density or size in either the metaphysis or diaphysis of rats after 10 weeks of STZ-induced T1D. In fact, contrary to prior results, both adipocyte density and size were substantially reduced in diabetic rats. Previous findings were observed in mice at 4- to 6-weeks post-induction; thus, our results may indicate a differential effect on bone marrow adiposity with sustained T1D that warrants further investigation.

In our study we also assessed the effect of chronic placement of a silicone sciatic nerve cuff for 12 weeks. We found that cuff placement contributed to independent changes in gait, tibial length, cortical bone and bone marrow adiposity. Cuff placement can be used as a model of chronic constriction injury (CCI) and neuropathic pain, but the largest inner diameter used in CCI models is approximately half of that used in our device (0.86 vs. 1.5 mm) (Mosconi and Kruger, 1996; Balasubramanyan et al., 2006; Austin et al., 2012). In addition, we did not see a unilateral increase in mechanical sensitivity induced by the placement of a sciatic nerve cuff, a typical indicator of neuropathy in CCI studies. Thus, it is unlikely that the effects on gait and bone were caused by overt nerve constriction. Instead, we anticipate that these results reflect local changes in nerve swelling, fibrosis and inflammation that occur after implantation of a local neuroprosthesis, contributing to the observed changes in gait and tibia length and unilateral reductions in cortical bone mass and bone marrow adiposity. Indeed, upon dissection, we observed that the sciatic nerve cuff implanted in all animals, regardless of glycemic condition or eStim, was enveloped in fibrotic tissue that fixed the cuff in place. To reduce activation of inflammatory responses and improve flexibility, more sophisticated designs for chronic neural implants are available. Extraneural electrodes like the one used herein are preferred (Günter et al., 2019), but helical or spiral cuff designs can provide more flexibility and relieve any tension or strain on the nerve (Naples et al., 1988; Christie et al., 2017). Noninvasive transcutaneous stimulation of the sciatic nerve is also possible, though reportedly ineffective for neuroregeneration (Baptista et al., 2008). Additional research is needed to investigate whether more flexible electrode designs would limit the undesirable effects on bone as observed in this study.

Beyond consideration of the effects of T1D and cuff placement, the primary goal of this study was to isolate the impact of chronic, intermittent eStim on nerve and bone health and on the progression of DPN. Contrary to our initial hypotheses, we did not observe an effect of eStim on muscle mass, bone quantity, or mechanical sensitivity in healthy animals or a rescue in those with T1D. However, we did find that treatment with eStim opposed unilateral cuff-induced effects on tibial length and gait. This suggests that intermittent application of a local bioelectric stimulus can counteract some of the negative side-effects of cuff placement. Previously, the device used herein was shown to evoke longitudinally consistent electromyogram amplitudes in the gluteal, tibialis anterior, and plantaris muscles over 14 weeks of implantation in healthy animals (Gamble et al., 2016). However, inclusion of additional functional assays such as *in vivo* joint torque measurements or more comprehensive gait analyses may provide additional insight into alterations in gait and bone caused by sciatic nerve cuffing and opposed by eStim. Local, stimulation-induced release of anabolic neuropeptides at the growth plate may also be involved in promoting unilateral tibial lengthening (Brazill et al., 2019). Additionally, bioelectric nerve stimulation has been shown to drive changes in muscular gene expression (Brownson et al., 1988); which may also underlie functional changes observed in gait independent of muscle mass. Future investigation is required to determine if eStim modulates endochondral ossification at the skeletal growth plate and aspects of muscle function, including underlying gene expression. In addition to the possible direct effects of eStim on the growth plate and muscle, inhibition of local neuroinflammation may also contribute to the outcomes. While we did observe fibrosis around the sciatic nerve cuff in all animals regardless of eStim, we acknowledge that it remains possible that eStim locally suppressed inflammation or fibrosis in more subtle ways than were grossly observable. Local neuroinflammatory suppression, in turn, may have indirectly contributed to the restoration of gait and tibial lengthening.

Contrary to its mild restorative effects, treatment with eStim unexpectedly resulted in a 1–2% bilateral reduction in cortical bone tissue mineral density, independent of T1D. The mechanism underlying this effect remains unknown. However, we hypothesize that nerve action potentials produced by the device used here may have travelled bidirectionally along the axon, resulting in bilateral reductions in skeletal mineralization through activation of central neural relays. Additional studies may address whether this effect was centrally mediated by eStim responses in the spinal cord or brain, or by treatment-induced systemic stress factors otherwise insufficient to alter body mass or blood glucose.

### Limitations

The eStim paradigm employed here was previously optimized to enhance neuroregeneration in a post-injury setting (Geremia et al., 2007; Singh et al., 2015; MacEwan et al., 2018), but it was unknown if this paradigm could be leveraged to prevent neuropathy or other musculoskeletal deficits associated with chronic metabolic disease. While it restored cuff-induced imbalances in gait and promoted unilateral tibial lengthening, eStim did not prevent T1D-associated mechanical allodynia, osteopenia, or sarcopenia. To the extent that our assays could measure, we did not observe a benefit of eStim therapy for nerve function in T1D animals. However, our interpretation is limited by assay specificity. Von Frey and gait analyses are indicative of large diameter sensory and/or motor fiber dysfunction. While our analyses show that eStim did not prevent large fiber neuropathy, it is left to future investigation to determine whether eStim can protect against small-fiber neuropathy. Our study was also limited to a discrete selection of eStim parameters and dosing regimens. It remains possible that increasing the number of weekly sessions or otherwise optimizing the eStim parameters could provide additional therapeutic benefit for nerve, muscle or bone. For example, in a more muscle-targeted approach, shorter, daily eStim bouts with implementation of a resting phase have been shown to protect bone in the context of disuse osteopenia (Lam and Qin, 2008; Lam et al., 2011; Tamaki et al., 2017). Additionally, peripheral pain is a common application of eStim clinically, in which parameters are typically optimized to block aberrant nerve activity, at either the peripheral or spinal level (Mobbs et al., 2007; Kumar et al., 2008). As such, it would be worthwhile to assess bone health in pre-clinical and clinical models that block neuronal activation, in addition to those designed for activation. Last, the present study was limited to male rats only. Sex differences have been reported in growth, metabolism, neuropathy, and sarcopenia in STZ-induced diabetic rodents (Vital et al., 2006; Pesaresi et al., 2011; Choi et al., 2013; Pesaresi et al., 2018; Virgen-Ortiz et al., 2018). Bone structural phenotypes are gender-independent in the STZ model (Martin and McCabe, 2007), but sexual dimorphism in eStim interaction with this phenotype may exist. Thus, it remains unknown whether the eStim regimen employed here would be therapeutic in female rats.

## Conclusion

Overall, the stimulation parameters and treatment regimen selected for this study were insufficient to prevent T1D-induced osteopenia, sarcopenia, and neuropathy. However, our results indicate that cuff device placement on peripheral nerves can unilaterally reduce cortical bone mass and tibial length and regulate bone marrow adiposity. In addition, while bioelectric nerve stimulation restored cuff-induced gait imbalances and unilaterally favored tibial lengthening, it also caused bilateral reductions in cortical bone mineral density. Altogether, this suggests that skeletal health should be monitored in long-term clinical applications of neuromodulation devices to prevent unintended consequences including decreased bone mineral density and increased fracture risk.

## Data Availability Statement

The original contributions presented in the study are included in the article/Supplementary Material, further inquiries can be directed to the corresponding author.

## Ethics Statement

The animal study was reviewed and approved by Institutional Animal Care and Use Committee, Washington University in Saint Louis, MO, United States.

## Author Contributions

AB: conceptualization, data curation, formal analysis, investigation, methodology, project administration, visualization, validation, writing, reviewing, and editing. IS: data curation, formal analysis, investigation, visualization, reviewing, and editing. XZ: data curation, formal analysis, investigation, methodology, validation, visualization, reviewing, and editing. KM: data curation, project administration, investigation, supervision, reviewing, and editing. YY: investigation, methodology, resources, supervision, reviewing, and editing. MM: conceptualization, methodology, resources, reviewing, and editing. ES: conceptualization, formal analysis, funding acquisition, methodology, project administration, resources, supervision, visualization, writing, reviewing, and editing. All authors contributed to the article and approved the submitted version.

## Conflict of Interest

The authors declare that the research was conducted in the absence of any commercial or financial relationships that could be construed as a potential conflict of interest.
